# Immune-related risk prognostic model for clear cell renal cell carcinoma: Implications for immunotherapy

**DOI:** 10.1097/MD.0000000000034786

**Published:** 2023-08-25

**Authors:** Ronghui Chen, Jun Wu, Shan Liu, Yefeng Sun, Guozhi Liu, Lin Zhang, Qing Yu, Juan Xu, Lingxin Meng

**Affiliations:** a Clinical Medical College of Weifang Medical University, Weifang, China; b Department of Oncology, People’s Hospital of Rizhao, Rizhao, China; c Department of Emergency, People’s Hospital of Rizhao, Rizhao, China; d Jining Medical University, Jining, China.

**Keywords:** clear cell renal cell carcinoma, immune microenvironment, prognostic model, tumor mutation burden

## Abstract

Clear cell renal cell carcinoma (ccRCC) is associated with complex immune interactions. We conducted a comprehensive analysis of immune-related differentially expressed genes in patients with ccRCC using data from The Cancer Genome Atlas and ImmPort databases. The immune-related differentially expressed genes underwent functional and pathway enrichment analysis, followed by COX regression combined with LASSO regression to construct an immune-related risk prognostic model. The model comprised 4 IRGs: CLDN4, SEMA3G, CAT, and UCN. Patients were stratified into high-risk and low-risk groups based on the median risk score, and the overall survival rate of the high-risk group was significantly lower than that of the low-risk group, confirming the reliability of the model from various perspectives. Further comparison of immune infiltration, tumor mutation load, and immunophenoscore (IPS) comparison between the 2 groups indicates that the high-risk group could potentially demonstrate a heightened sensitivity towards immunotherapy checkpoints PD-1, CTLA-4, IL-6, and LAG3 in ccRCC patients. The proposed model not only applies to ccRCC but also shows potential in developing into a prognostic model for renal cancer, thus introducing a novel approach for personalized immunotherapy in ccRCC.

## 1. Introduction

Kidney cancer, including clear cell renal cell carcinoma (ccRCC), is a prevalent urological malignancy with over 430,000 new cases reported in 2020.^[1]^ Approximately one-third of ccRCC patients have metastases at the time of initial diagnosis, and about 25% of patients with limited ccRCC experience recurrent metastases after radical surgery, often resulting in high mortality rates.^[2–4]^ Immunotherapy has emerged as a promising alternative to chemotherapy and targeted therapy for ccRCC, resulting in noteworthy enhancements in the prognosis of patients with metastatic ccRCC.^[5–7]^ However, the intricate and diverse tumor immune microenvironment gives rise to substantial heterogeneity in the prognosis of patients undergoing immunotherapy. Hence, it has become an inevitable trend to guide clinical treatment by analyzing the tumor immune infiltration microenvironment and constructing a prognosis model.

The tumor microenvironment (TME) is micro-ecosystem comprising tumor cells, immune cells, and secreted small molecules.^[8]^ Immune cells can both suppress tumor growth by clearing tumor cells and protect tumor cell subsets from developing drug resistance to immunotherapy.^[9–11]^ Tumor angiogenesis, invasion ability, immune escape, and immunosuppression, among other tumor lesion processes, are also closely related to the tumor microenvironment.^[12,13]^ Therefore, research on immunotherapy targeting the tumor microenvironment has become an important direction of study. Tumor mutation burden (TMB) was defined as the total count of coding mutations in somatic cells.^[14]^ Neoplasms types with high TMB promote the generation and delivery of tumor-specific antigens, enhancing the immune response. TMB has great potential in predicting immunotherapy outcomes.^[14–16]^

ccRCC has demonstrated the greatest level of immune infiltration among diverse cancer types, as evidenced by transcriptomic studies.^[17]^ Furthermore, the unique immune response-related gene expression patterns displayed by ccRCC, when contrasted with other RCC subtypes, have garnered notable interest.^[18]^ Immune checkpoints not only modulate the tumor’s responsiveness to immune checkpoint inhibitors, but their association with survival prognosis also varies in accordance with different degrees of TME infiltration.^[19,20]^ A study by Lucia et al^[21]^ underscored the effectiveness of dual immunotherapy for treating intermediate-to-poor-risk metastatic renal cell carcinoma. ccRCC diverges from other tumors in that it exhibits extensive immune infiltration, coupled with high levels of TMB. Elevated TMB levels often denote enhanced sensitivity to Immune Checkpoint Inhibitors, with recent studies drawing a notable correlation between high TMB levels in ccRCC and a significant drop in survival rates.^[22,23]^ However, the interaction between ccRCC-associated immune checkpoints, TMB, TME, and survival rate creates a seemingly paradoxical and unclear relationship.

**Figure 1.**
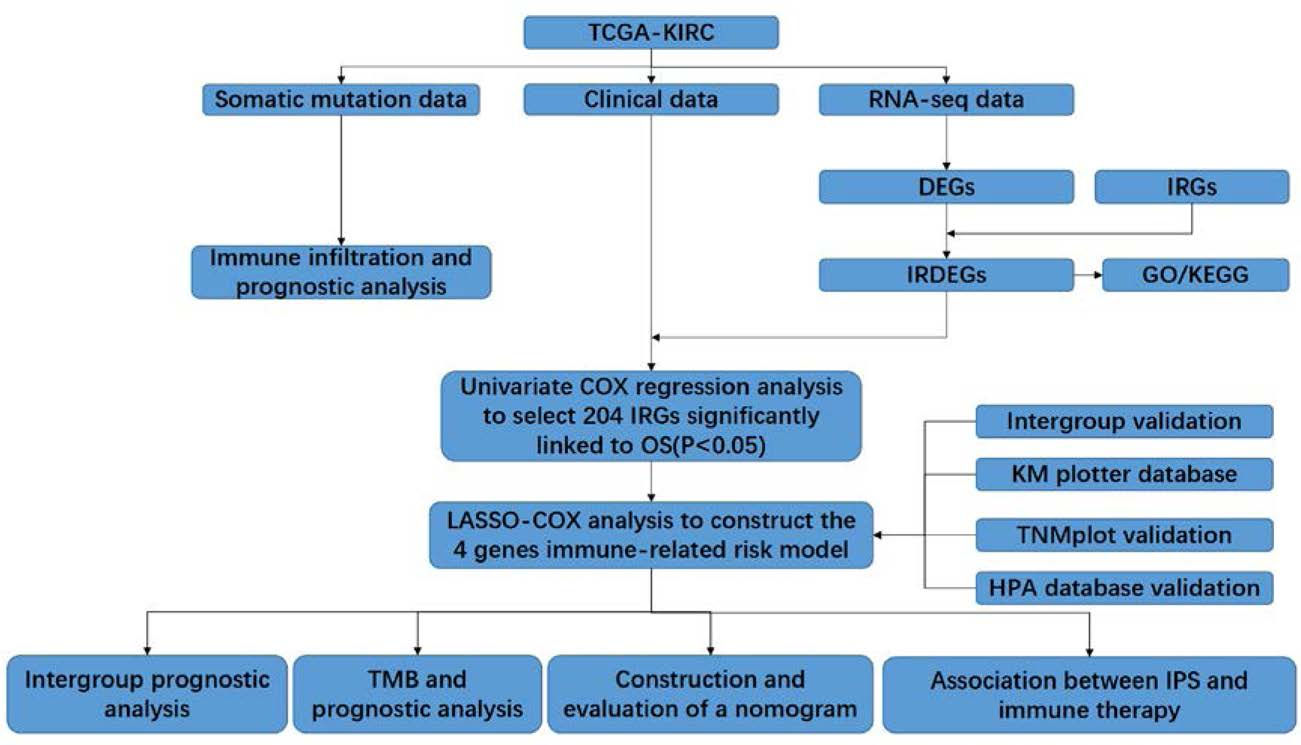
Flowchart of the study. DEG = differentially expressed gene, GO = gene ontology, IRDEG = immune-related differentially expressed gene, IRG = immune-related gene, KEGG = Kyoto Encyclopedia of Genes and Genomes, LASSO = least absolute shrinkage and selection operator, OS = overall survival, TCGA = The Cancer Genome Atlas, TMB = tumor mutation burden.

The aim of our research is to construct a prognostic model, grounded in immune-related genes (IRGs), which possesses the capacity for both analyzing tumor microenvironment infiltration and prognosticating TMB, thereby informing the application of immune inhibitors. We envisage these IRGs, along with the prognostic model, offering indispensable insights into clinical treatment strategies and prognosis prediction. The research methodology is delineated in Figure 1.

## 2. Methods

### 2.1. Data preprocessing

We obtained the RNA sequencing data for ccRCC and normal tissue from The Cancer Genome Atlas (TCGA) database (https://portal.gdc.cancer.gov)^[24]^ project TCGA-KIRC. The database also includes clinical characteristics data for 537 patients. For transcriptome profiling, a Perl script^[25]^ was used to convert Entrez IDs to the corresponding official gene names, and genes with zero expression in the samples were removed. Patient clinical characteristics, such as survival outcome, age, sex, tumor stage, and histological grade, were obtained from an appropriate data source.^[26]^ IRGs were retrieved from the immunology database ImmPort (https://www.immport.org).^[27,28]^

### 2.2. Identification of IRDEGs

Initially, differential gene expression analysis was conducted between ccRCC samples and normal kidney tissues (FDR < 0.05, |log2FC|>1). Heatmaps and volcano plots were generated using the “ggplot2” package.^[29]^ The intersected genes between the screened ccRCC differentially expressed genes (DEGs) and immune genes were visualized using the “venn” package^[30]^ through a Venn diagram, resulting in the identification of immune-related differentially expressed genes (IRDEGs) in ccRCC.

### 2.3. Gene ontology (GO) and Kyoto encyclopedia of genes and genomes (KEGG) enrichment analysis

Functional and pathway enrichment analysis of IRDEGs was conducted using the “clusterProfiler” package.^[29]^ GO is an internationally recognized classification system for gene function that enhances our understanding of biological functions of genes. Additionally, pathway enrichment analysis was performed using the KEGG database.

### 2.4. Construction of immune-related risk prognostic model

#### 2.4.1. Univariate COX regression analysis.

Clinical data underwent preprocessing, which involved excluding samples with a survival time of less than 30 days and removing duplicates from the expression matrix. Univariate COX regression analysis was performed using the “survival” package to evaluate the association between immune genes and overall survival (OS) of patients (*P* < .05).

#### 2.4.2. LASSO regression analysis.

By adjusting the regression coefficient of the self-factor, it reduces overfitting of the data and identifies relatively important factors. The IRDEGs screened by single-factor COX regression were analyzed using the “corrplot” package for least absolute shrinkage and selection operator (LASSO) regression, and the minimum λ value was calculated as the reference value for the best factor in the model. Subsequently, the IRDEGs with higher correlation with the prognosis of ccRCC patients were obtained.

#### 2.4.3. Construction of ccRCC immune-related risk prognostic model.

Next, the obtained genes were subjected to multivariate COX regression to calculate the regression coefficient of each gene and establish the optimal IRDEGs -related risk model. The risk score was calculated using the formula:


$$\text{Risk Score}\sum_{i=1}^{n}\text{Xi}\times \text{Coef}$$

where Coef represents the risk regression coefficient of the gene, Xi represents the expression level of IRDEGs, and *n* is the number of genes screened out.

### 2.5. Multidimensional verification

The patients were categorized into 3 groups: a training group, a test group, and an overall group. Subsequently, the patients were further divided into high- and low-risk groups based on the median value of the model risk score. Kaplan–Meier survival curve was performed using the “survival” and “Survminer” packages to compare the differences in survival prognosis between the high- and low-risk groups in the training, test, and overall groups. Additionally, the “timeROC” package was employed to generate time-dependent ROC curves and calculate the area under the curve to assess the reliability of the model. The prognostic significance of IRDEGs was evaluated using the KM plotter database (https://kmplot.com/analysis/). Leveraging resources such as NCBI-GEO, TCGA, TARGET, and the GTEx Database, the TNMplot (https://tnmplot.com/analysis/) construct provides a visual representation of IRDEGs and their associated signatures within normal kidney tissue and renal carcinoma. The results of immunohistochemical pathology of IRDEGs were assessed using HPA database (https://www.proteinatlas.org/).

### 2.6. Independent prognostic analysis of risk scores and construction of nomogram

The clinical characteristics of the patients were obtained and combined with the risk scores of the high- and low-risk groups for conducting univariate and multivariate COX regression analyses. The clinical characteristics were utilized as independent variables, OS was considered as the dependent variable, and hazard ratio (HR), 95% confidence interval, and *P* value were calculated. The risk score was incorporated in the multivariate analysis to ascertain its independent prognostic significance (*P* < .05). To investigate potential differences in the risk scores of patients across various clinical parameters, a boxplot of the clinical information for all patients was created using the “ggpubr” and “limma” packages. The significant prognostic variables identified through univariate and multivariate COX regression were used to construct a nomogram, which was subsequently utilized for predicting outcomes. This approach allowed for a more accurate prediction of outcomes by incorporating multiple factors into the analysis.

### 2.7. Differential analysis of tumor immune cell infiltration and survival analysis

The proportions of immune cells in the high- and low- risk groups were calculated, and the “vioplot” package was used to create violin plots for visual analysis of the differences in immune cell content. Additionally, survival curve analysis was performed to compare the prognosis between the 2 groups.

### 2.8. Survival analysis of TMB in high- and low-risk groups

The “maftools” package^[31]^ was utilized to analyze and describe the somatic mutation landscape in the high- and low-risk groups, using the somatic mutation data of ccRCC patients from the TCGA database. The association between the high- and low-risk groups and TMB as well as survival prognosis was assessed.

### 2.9. IPS and immune checkpoints analysis in high- and low-risk

The study conducted an examination of ccRCC patients, focusing on the sensitivities to 4 immune checkpoints: Programmed cell death protein 1 (PD-1 or PDCD1), Cytotoxic T-Lymphocyte Antigen 4 (CTLA-4), Interleukin 6 (IL-6), and Lymphocyte Activation 3 (LAG3). The “limma” package algorithm was utilized to perform differential expression analysis of PD-1 and CTLA-4 genes between the high- and low-risk groups. the Immunophenoscore (IPS) was retrieved from TCIA database (https://tcia.at/). A negative correlation with IPS indicates poor efficacy of immunotherapy, while a positive correlation with IPS suggests that it can be used as a predictor of good prognosis for immunotherapy.^[32]^

## 3. Result

### 3.1. Identification and functional enrichment analysis of IRDEGs

**Figure 2.**
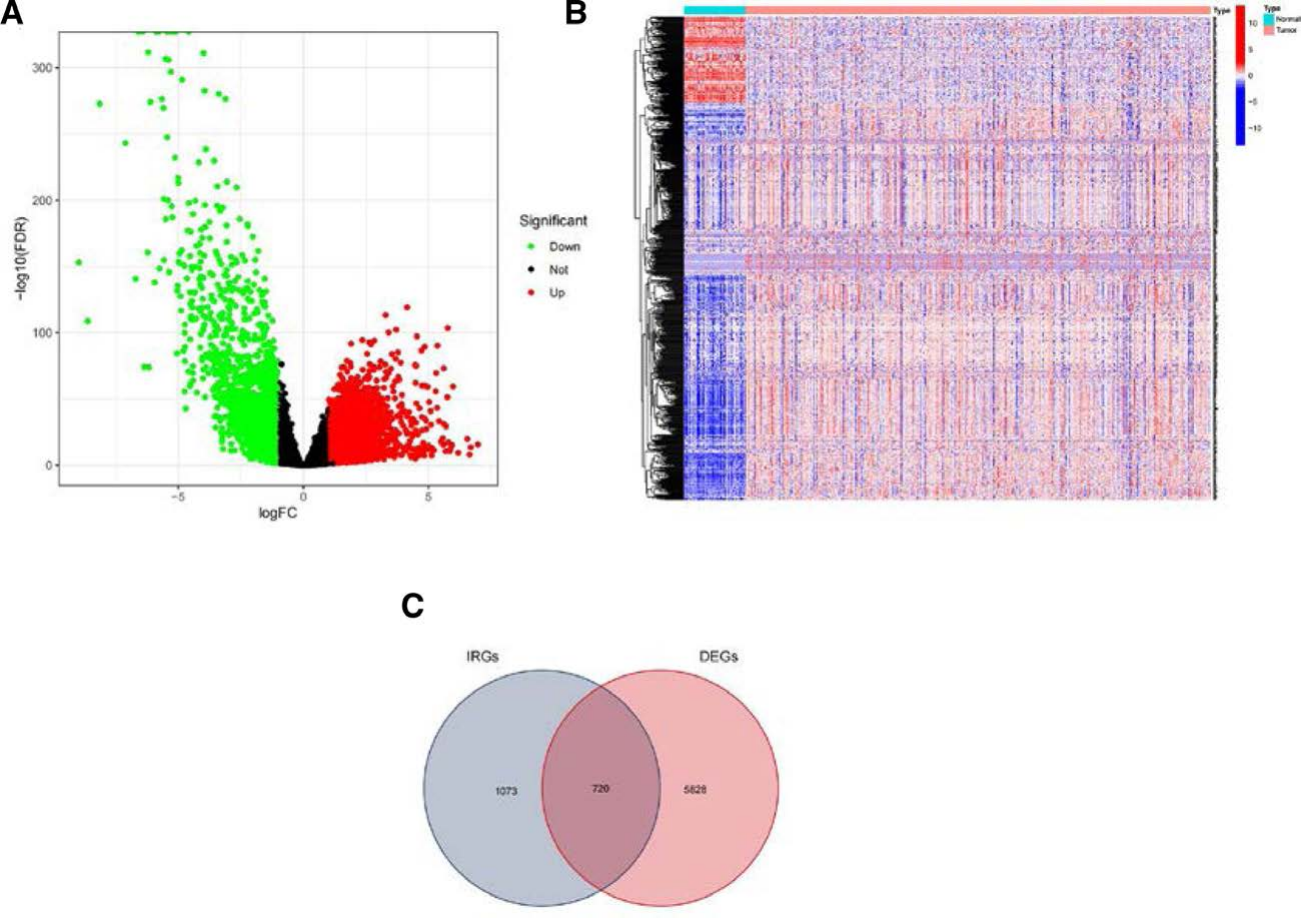
Identify the relevant genes for ccRCC in the TCGA database. Volcano plot (A) and heatmaps (B) of differentially expressed genes between ccRCC and normal kidney tissue, and Venn diagram (C) of IRDEGs. ccRCC = clear cell renal cell carcinoma, IRDEG = immune-related differentially expressed gene, TCGA = The Cancer Genome Atlas.

**Figure 3.**
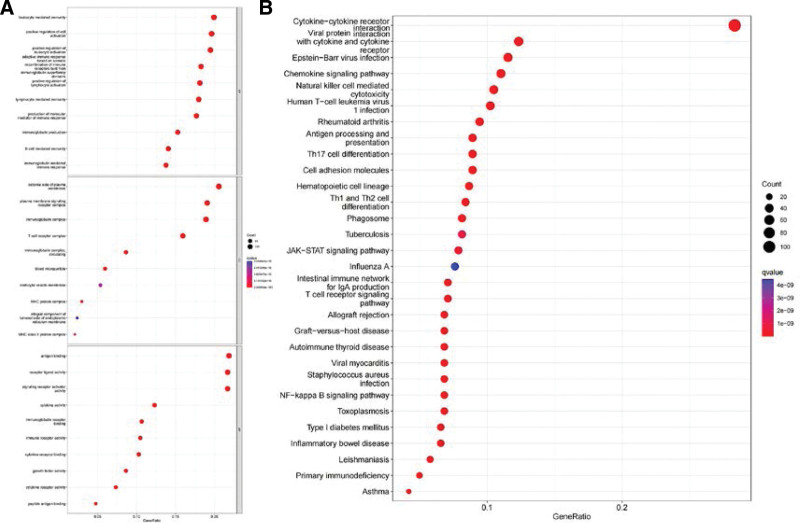
Functional enrichment analysis of immune related IRDEGs in ccRCC. (A) Results of gene ontology (GO) analysis: molecular function (MF), cellular component (CC) and biological process (BP). (B) Results of Kyoto Encyclopedia of Genes and Genomes (KEGG) analysis. ccRCC = clear cell renal cell carcinoma, IRDEG = immune-related differentially expressed gene.

A volcano plot (Fig. 2A) was generated based on the analysis of differential gene expression in 542 cases of ccRCC tissues and 72 cases of normal kidney tissues, revealing that 4638 genes were up-regulated (depicted in red) and 1910 genes were down-regulated (depicted in green). The gene clustering heatmap (Fig. 2B) of ccRCC and normal tissues shows the gene distribution trend of tissue types. Subsequently, 720 IRDEGs were identified (Fig. 2C) by identifying the intersection of DEGs and immune genes. Functional enrichment analysis using Gene Ontology (GO) for the IRDEGs showed enrichment in biological processes (BP) associated with leukocyte-mediated immunity and regulation of activation, cellular components (CC) related to plasma membrane conduction and receptor complexes, and molecular functions (MF) associated with antigen-receptor binding activity (Fig. 3A). The cytokine-cytokine receptor interaction pathway was the most significantly enriched KEGG pathway (Fig. 3B).

### 3.2. Construction of immune-related risk prognostic model

**Figure 4.**
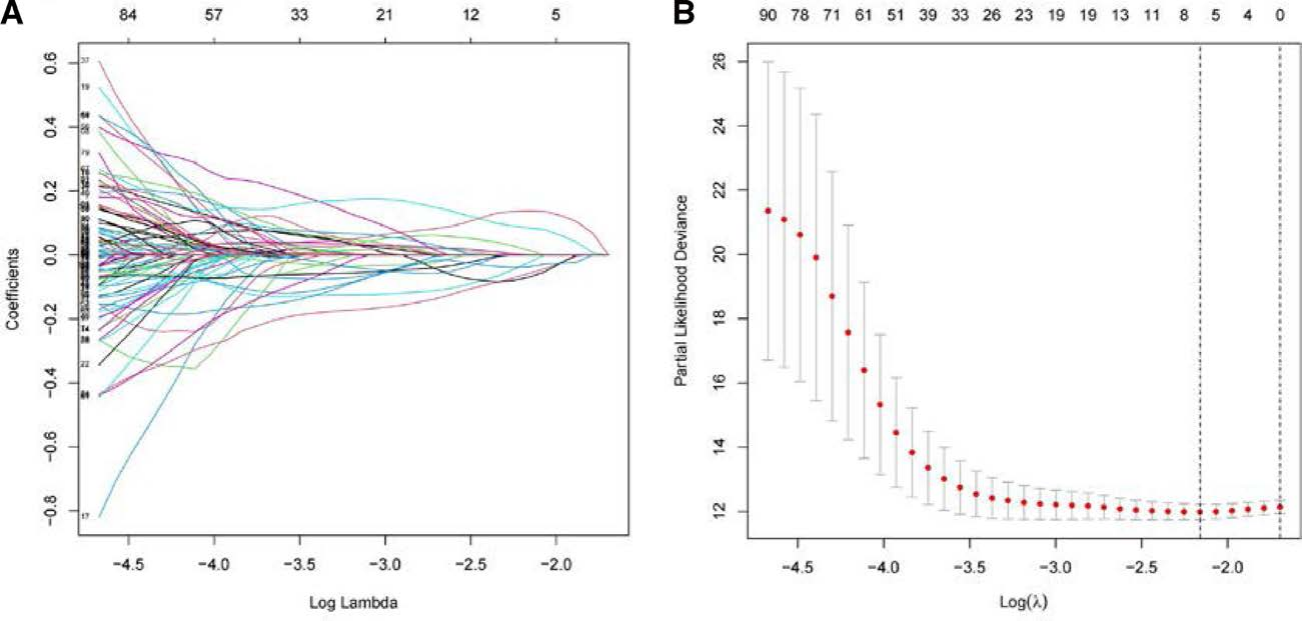
Construction of the immune-related genes risk model. (A) Cross-validation of parameter selection in the LASSO model (cvfit curve) and (B) the LASSO regression coefficient spectrum (lambda curve). LASSO = least absolute shrinkage and selection operator.

Following the univariate COX regression analysis, 204 IRGs were found to exhibit significant correlation with OS at a significance level of *P* < .05 (Table 1). Subsequent screening using LASSO regression, which involves the tuning of the lambda parameter to control the complexity of the model, was conducted. The optimal lambda value was determined by cvfit through cross-validation (Fig. 4A), as represented in the lambda curves (Fig. 4B). This lambda value minimizes the cross-validation error, thereby striking a balance between model simplicity and predictive power. Following this optimization process, along with the application of multivariate COX regression, 4 genes were identified. CLDN4, SEMA3G, and catalase (CAT) were identified as independent prognostic factors among these genes, whereas urocortin (UCN) was not (Table 2). Despite the multivariate regression results for UCN indicating a *P* value greater than .05, this merely suggests that the UCN index should not serve as an independent prognostic factor. Nonetheless, this observation does not preclude the integration of UCN with other variables in the construction of a comprehensive prognostic model. Subsequently, an immune-related risk prognosis model was established utilizing the following equation: Risk Scores = (−0.310) × CLDN4 + (−0.291) × SEMA3G + (−0.286) × CAT + (0.230) × UCN.

**Table 1:** Univariate COX regression of immune-associated genes and overall survival.

Gene	COEF	HR	HR.95L	HR.95H
B2M	0.606838974	0.469411031	0.78450125	0.000137581
HLA-C	0.722144077	0.553498647	0.942174061	0.016443737
HLA-DRA	0.849197278	0.724161324	0.995822331	0.044274848
HLA-G	0.880441574	0.796582111	0.973129267	0.012655191
HLA-H	0.747774525	0.606298219	0.922263538	0.006603256
MICB	1.448589478	1.065107053	1.970141377	0.01817772
RELB	1.430488141	1.010696579	2.024639604	0.043383833
IFI30	1.554986862	1.239233653	1.951193091	0.000137761
PDIA2	1.240887154	1.068570958	1.440990808	0.004663084
HAMP	1.478304673	1.251555816	1.746134433	4.20E-06
SLPI	1.101099575	1.041200674	1.164444381	0.000738975
CXCL5	1.184085936	1.095206947	1.280177695	2.19E-05
CXCL1	1.172666895	1.076476426	1.277452635	0.000264755
CXCL13	1.179551959	1.074411784	1.294980979	0.000526842
CXCL2	1.207768933	1.082016976	1.348135776	0.000765007
XCL1	1.218432268	1.022279032	1.452223069	0.027384018
TMSB10	1.351893597	1.085068233	1.684333061	0.007193172
S100A8	1.153800217	1.006744024	1.322337068	0.039725882
CCR10	1.47943283	1.217188412	1.798178059	8.34E-05
MMP12	1.210495228	1.08529538	1.350138152	0.000604954
SERPIND1	1.150165	1.01473303	1.303672482	0.028613113
LMBR1L	2.060767947	1.478323036	2.872690492	1.99E-05
IL6	1.173938011	1.084868899	1.270319809	6.79E-05
TGFB1	1.373263183	1.073502092	1.756728546	0.01158786
MMP9	1.126912595	1.025856597	1.237923507	0.012684671
APOBEC3G	1.244989936	1.008183668	1.537418221	0.041782464
NOD2	1.556033683	1.198416726	2.020366347	0.000905153
PLAU	1.290176903	1.11015254	1.499394345	0.000891154
PAEP	1.145551715	1.075711399	1.219926398	2.30E-05
LBP	1.083495144	1.024831524	1.1455168	0.004748451
NOX4	0.758902907	0.637581454	0.903309872	0.001908259
FABP5	1.397678381	1.146472113	1.70392706	0.000925698
OASL	1.245525434	1.026822719	1.510809587	0.025837625
CETP	0.793683376	0.630375816	0.999298016	0.049305711
C8G	1.199845079	1.032477176	1.394343863	0.01745713
APOD	1.164700593	1.017853616	1.33273336	0.026601028
PML	1.713005586	1.066107374	2.752432082	0.026112998
ISG20	1.400568191	1.096806737	1.78845661	0.006918088
TLR3	0.763998633	0.660457289	0.88377238	0.00029143
ISG15	1.356315274	1.11466858	1.650348056	0.002332005
TFR2	1.180472684	1.026452743	1.357603422	0.020019122
LYZ	0.872557898	0.782892555	0.972492687	0.01373455
APOM	0.842791289	0.755734808	0.939876196	0.002107573
BPHL	0.447466511	0.326930359	0.61244321	5.12E-07
WNT5A	1.475435514	1.196760104	1.81900278	0.000270853
SLC11A1	1.506862756	1.251774003	1.813933953	1.47E-05
TNFRSF10B	1.475291932	1.078437371	2.018185148	0.015003287
APOBEC3H	1.387575784	1.13564592	1.695393363	0.001353923
TMPRSS6	1.240377831	1.033243224	1.48903678	0.02084587
MARCO	1.14825215	1.024963421	1.286370784	0.017058941
TNFSF11	1.288153203	1.029354026	1.612019416	0.026911743
KLRK1	1.481351189	1.048694654	2.092507419	0.025763567
CLDN4	0.627423197	0.529845136	0.742971562	6.49E-08
IRF7	1.542614837	1.23869655	1.921100479	0.000107922
LTB4R	1.615406694	1.328013268	1.964994516	1.60E-06
SYTL1	1.376188306	1.14241659	1.657796525	0.000774682
APOBEC3C	1.285846746	1.044865911	1.582405777	0.017574616
CD14	1.208221704	1.007762648	1.448555063	0.04100247
MASP1	0.821089489	0.723477587	0.93187123	0.002268112
NDRG1	0.755379746	0.636325016	0.896709301	0.001346671
IRF9	1.505438947	1.22106393	1.856042396	0.000128306
HMOX1	0.844112269	0.733364407	0.971584544	0.018191848
AQP9	1.133727717	1.029749727	1.248204784	0.01054977
BIRC5	1.566723353	1.323395166	1.854791471	1.85E-07
VTN	1.168556681	1.060562421	1.287547709	0.001641556
VIM	1.37844531	1.091464004	1.740883313	0.00704323
AGER	1.504476329	1.25196522	1.807916855	1.32E-05
ACO1	0.547889016	0.403560415	0.743835031	0.000114769
CCL11	1.31477944	1.090199072	1.585623232	0.004188262
XCL2	1.228299364	1.045159777	1.443529843	0.01255536
CDH1	0.794980451	0.688043652	0.918537532	0.001853288
CRP	1.132235517	1.031521355	1.242783059	0.008977765
RNASE2	1.362855566	1.160357743	1.600691947	0.000161826
CD79A	1.113270346	1.000254933	1.239054987	0.049456954
VAV3	0.631000894	0.525172772	0.758154552	8.84E-07
CHP1	0.654470933	0.454727151	0.941954315	0.02249906
CARD11	1.214795289	1.055912915	1.397584567	0.006514247
CD72	1.29506104	1.083024191	1.548610927	0.004593447
LILRB3	1.541730645	1.238551582	1.919123446	0.000106629
FCGR2B	1.246276864	1.033763411	1.502477265	0.020994527
IGHA1	1.099465315	1.013626096	1.192573854	0.022237778
IGHD2-2	1.191842791	1.032686612	1.375527893	0.016405566
IGHD3-3	1.129936437	1.002791265	1.273202505	0.044885165
IGHG1	1.106033579	1.038828236	1.177586665	0.001627185
IGHG2	1.095245569	1.016022098	1.180646424	0.017554357
IGHG3	1.103854608	1.031893346	1.180834241	0.004069139
IGHG4	1.085008391	1.012419015	1.162802348	0.020926075
IGHJ5	1.178819023	1.072065184	1.296203168	0.000681937
IGHV1-24	1.115862078	1.042797098	1.194046453	0.001509656
IGHV1-3	1.076825821	1.000499448	1.158975001	0.04846416
IGHV1-46	1.086018323	1.002345933	1.176675397	0.043669876
IGHV1-69	1.084270917	1.005950506	1.16868913	0.034424944
IGHV3-30	1.088744051	1.009432059	1.174287658	0.027578577
IGHV4-59	1.086909704	1.005630918	1.17475774	0.035591708
IGKC	1.10383725	1.02984228	1.183148817	0.00526132
IGKJ3	1.135804548	1.034266846	1.247310572	0.007696099
IGKJ4	1.100957332	1.005092142	1.205966097	0.038523623
IGKJ5	1.112979271	1.022538514	1.211419268	0.01330875
IGKV1-5	1.080105493	1.004835576	1.161013706	0.036541277
IGKV1D-12	1.164922225	1.00805749	1.346196822	0.038572487
IGKV2-28	1.145758384	1.039491315	1.262889122	0.006146365
IGKV3-11	1.075324013	1.001007048	1.155158433	0.046865811
IGKV3-15	1.09683418	1.021920477	1.177239566	0.010445915
IGKV3D-11	1.113605525	1.005826612	1.232933442	0.038281664
IGKV5-2	1.121092852	1.030079079	1.220148247	0.00814524
IGLC2	1.119293828	1.041551039	1.202839444	0.002152174
IGLC3	1.119296639	1.043387561	1.200728295	0.001659052
IGLC6	1.277045748	1.12320505	1.451957362	0.000188462
IGLC7	1.088453649	1.000100108	1.184612758	0.049729717
IGLV1-44	1.081193711	1.004526232	1.163712608	0.03749733
IGLV2-14	1.083046899	1.00334149	1.169084104	0.040805966
IGLV3-1	1.079003052	1.007084188	1.156057855	0.03073043
IGLV3-10	1.094423212	1.021305303	1.172775823	0.01054196
IGLV3-16	1.157381543	1.014133482	1.320863633	0.030147002
IGLV3-19	1.085259393	1.016486652	1.158685111	0.014304617
IGLV3-21	1.103110056	1.031977767	1.179145359	0.003907713
IGLV3-27	1.130063901	1.03373932	1.235364076	0.007145986
IGLV5-37	1.220724935	1.084703802	1.373803027	0.000936684
IGLV9-49	1.085404388	1.007917482	1.168848351	0.030108994
EDN1	0.869802087	0.771524719	0.980598095	0.022593139
SAA1	1.118022775	1.066520972	1.172011576	3.54E-06
SAA2	1.14605921	1.054822564	1.245187349	0.001277608
SEMA3F	0.750481476	0.597731785	0.942266181	0.013431226
SEMA3G	0.679379029	0.592220177	0.779365316	3.42E-08
SEMA5A	0.698731632	0.571820665	0.853809462	0.000455942
SEMA5B	0.873242292	0.774398455	0.984702508	0.027002335
SEMA6A	0.808174866	0.688819206	0.948211967	0.008996433
SEMA6D	0.715574596	0.568230474	0.901125559	0.00444112
TYMP	1.273825802	1.02840637	1.577812256	0.026658253
LTB4R2	1.857640489	1.330324503	2.593974762	0.000277495
PLAUR	1.462119425	1.229602094	1.738605702	1.72E-05
PLXNA3	1.495279591	1.120219662	1.995913063	0.006324987
PLXNA4	1.369260241	1.102572798	1.700453349	0.004462877
PLXNB3	1.644281418	1.403956791	1.925744009	6.89E-10
AMH	1.770477336	1.457798537	2.150221664	8.33E-09
BMP1	1.667143846	1.326416541	2.095396519	1.18E-05
BMP5	0.749302523	0.585270711	0.959306968	0.022048637
BMP6	0.808872893	0.660675735	0.990312377	0.039952224
BMP8A	1.446766395	1.166117816	1.794958428	0.000788784
BTC	0.762228779	0.603560413	0.962609043	0.022609323
CAT	0.501606472	0.396189622	0.635072296	9.94E-09
CMTM3	1.58217753	1.272291447	1.967541119	3.70E-05
CMTM4	0.756716223	0.605815126	0.945204927	0.01402722
EBI3	1.265433808	1.041949057	1.53685318	0.017575808
ESM1	0.852826614	0.755880225	0.962206987	0.009718308
FGF1	0.795021314	0.647356887	0.976368527	0.028663203
GDF6	0.845168728	0.747958435	0.9550132	0.006969302
GDF7	0.757345096	0.59235894	0.968283848	0.026619118
GNRH1	1.593382356	1.300407002	1.952363629	6.99E-06
GREM1	1.241016012	1.03074112	1.494187736	0.022630537
IL11	1.532718769	1.183413914	1.985126925	0.001211665
INHBE	1.201753982	1.053327135	1.371096012	0.006287654
KITLG	0.733416359	0.609253589	0.882882867	0.001052052
KL	0.762520295	0.684885579	0.848955237	7.46E-07
MDK	1.238484126	1.102351151	1.391428611	0.000318019
NGF	1.211447423	1.022322659	1.435559357	0.02676777
OSM	1.177106583	1.009606372	1.372396161	0.037338963
PDGFD	0.707889931	0.616561142	0.812746896	9.49E-07
PDGFRL	1.367727919	1.214424032	1.540384257	2.43E-07
PGF	1.128696498	1.019137066	1.250033805	0.020134476
PTHLH	1.11705948	1.034831939	1.205820804	0.004544929
RETN	1.2264677	1.019388666	1.475612854	0.03050423
SCG2	1.130539957	1.021070111	1.251746164	0.01821394
TNFSF13B	1.338331084	1.117648313	1.602588283	0.00152567
TNFSF14	1.41044616	1.218097752	1.633168081	4.28E-06
TNFSF15	0.723402892	0.53673601	0.974989073	0.033478478
UCN	1.846760798	1.506291165	2.264187378	3.64E-09
ANGPTL1	0.795532399	0.663664439	0.953602093	0.013370416
ANGPTL3	0.793165537	0.699922878	0.898829839	0.000281713
APLNR	0.793142291	0.700735116	0.897735363	0.000245524
AVPR1B	0.813900682	0.706877377	0.937127628	0.004200192
CSF3R	1.258003133	1.029717527	1.536899043	0.024665512
ESRRG	0.723817751	0.605112354	0.865809687	0.000405349
FLT1	0.794140018	0.700300503	0.900553927	0.00032747
FLT4	0.797568595	0.668997213	0.950849496	0.011672364
IL15RA	1.606548596	1.23051265	2.097498462	0.000492861
IL1R2	1.12626317	1.025918886	1.236422047	0.012510309
IL20RB	1.175722972	1.096563903	1.260596399	5.31E-06
IL21R	1.339539915	1.059271268	1.693963802	0.014657673
IL2RA	1.285817059	1.102479174	1.499643303	0.001360131
IL2RG	1.20802688	1.033090779	1.412585392	0.017891848
IL4R	1.459429418	1.026585774	2.074774733	0.035191998
INSR	0.703309663	0.579180508	0.854042004	0.000381667
KDR	0.747540902	0.66444391	0.84103021	1.30E-06
LGR4	0.564926014	0.44586602	0.715778702	2.26E-06
MC1R	1.722201519	1.277060654	2.322503683	0.00036685
NR3C2	0.600586325	0.49545055	0.728032158	2.07E-07
NRP1	0.786929102	0.638915308	0.96923239	0.02420118
PPARA	0.540241088	0.398129478	0.733079186	7.69E-05
PTGER1	1.265678092	1.051713486	1.523172473	0.012645458
PTH1R	0.821184129	0.721071922	0.935195719	0.002977754
SORT1	0.784418195	0.6165606	0.997974739	0.048102016
TACR1	0.655210431	0.475788174	0.902293778	0.009605206
TEK	0.658255287	0.57229669	0.75712481	4.72E-09
TGFBR3	0.607919873	0.480840393	0.768584705	3.19E-05
THRB	0.650092737	0.511872157	0.82563695	0.000414066
TNFRSF17	1.204972294	1.041248408	1.394439807	0.012333726
TNFRSF18	1.483115346	1.243237431	1.76927679	1.19E-05
TNFRSF25	1.460409567	1.204070681	1.771321348	0.000120165
ZAP70	1.303281028	1.064394795	1.59578142	0.0103459
SHC3	1.508754506	1.129499974	2.015352115	0.005363104
CTLA4	1.282237331	1.055410204	1.557813792	0.01231754
TRAJ2	1.240238686	1.016748211	1.512854393	0.033686557
TRAJ31	1.358308571	1.122154426	1.644160671	0.001674087

The comparison among groups was statistically significant, *P* < .05.

**Table 2:** Multivariate COX regression of immune-associated genes and overall survival.

Gene	COEF	HR	HR.95L	HR.95H	*P* value
CLDN4	−0.310	0.7334	0.612	0.879	<.001
SEMA3G	−0.291	0.747	0.646	0.864	<.001
CAT	−0.286	0.752	0.568	0.995	.046
UCN	0.230	1.259	0.981	1.616	.070

The comparison among groups was statistically significant, *P* < .05.

### 3.3. Validation of immune-related risk prognostic model

**Figure 5.**
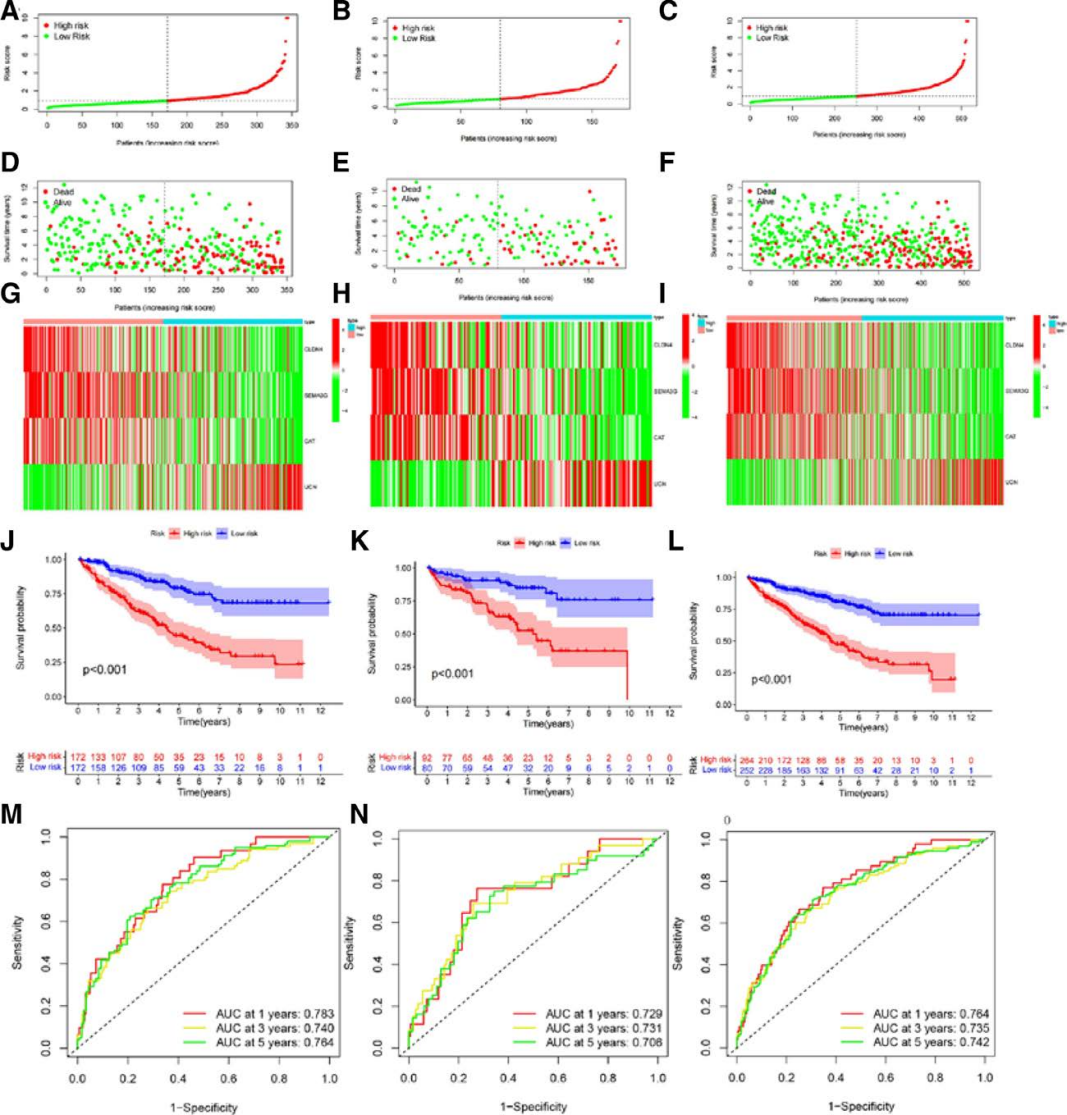
Build and validate a risk model for immune-related genes in the training, testing, and overall groups. (A–I) The distribution of immune-related risk scores, as well as the overall survival status, risk score distribution, and key gene associations (red for positive correlation, green for negative correlation) in the training, testing, and overall groups. (J–L) Kaplan–Meier survival curves represent the survival status and time of the training group, validation group, and overall group. (M–O) Receiver operating characteristic curve (ROC) curves validate the predictive accuracy of the immune-related risk model for predicting 1, 3, and 5-year overall survival (OS) in the training, testing, and overall groups. AUC = area under the curve.

**Figure 6.**
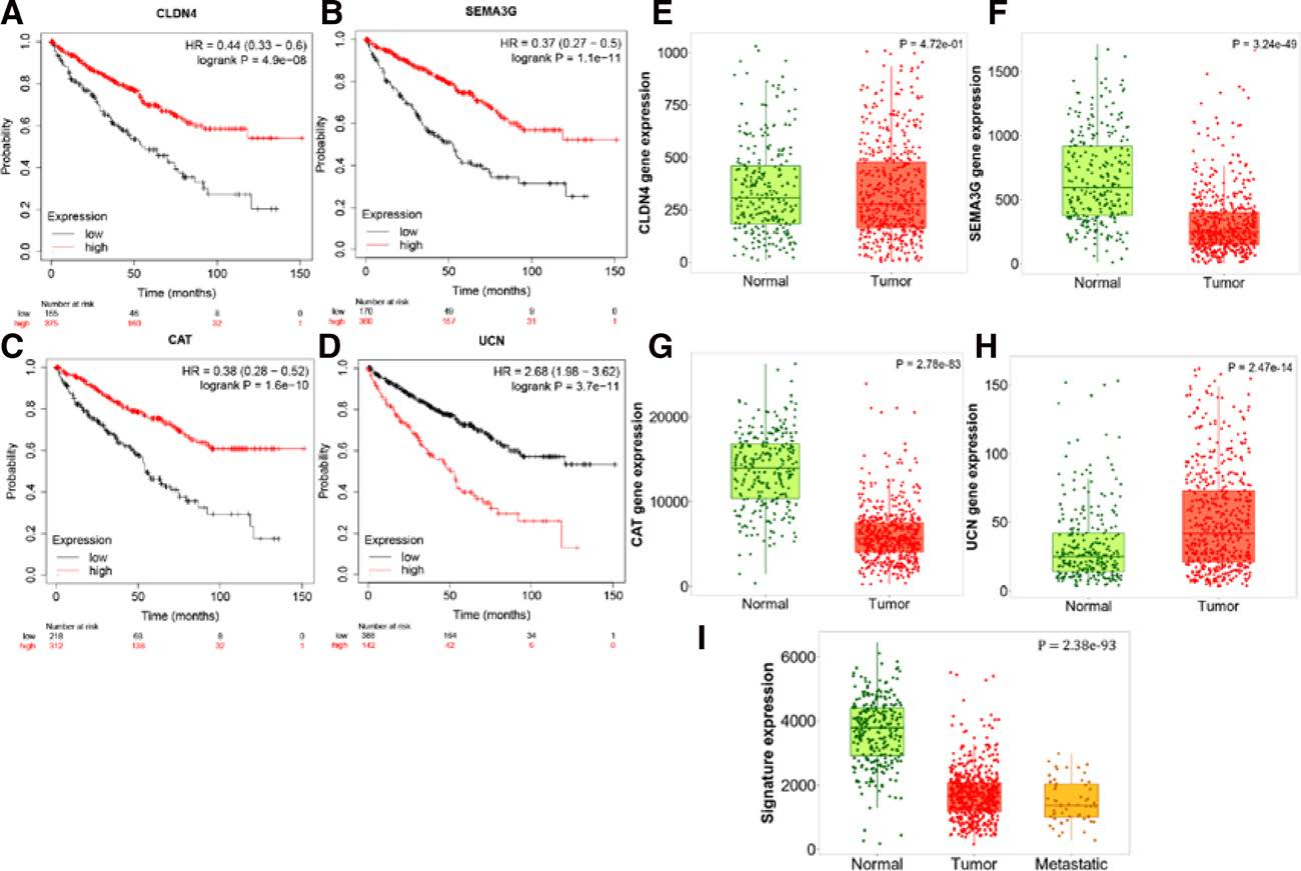
Validation of IRDEGs as potential prognostic biomarkers was performed using an external dataset. (A–D) The overall survival (OS) analysis of CLDN4, SEMA3G, CAT, and UCN in the ccRCC dataset (Kaplan–Meier Plotter). (E–I) Differential expression results of CLDN4, SEMA3G, CAT, UCN in the gene chip dataset of normal kidney-renal cancer (TNMplot). CAT = catalase, ccRCC = clear cell renal cell carcinoma, CLDN4 = claudin 4, IRDEG = immune-related differentially expressed gene, SEMA3G = semaphorin 3G, UCN = urocortin.

**Figure 7.**
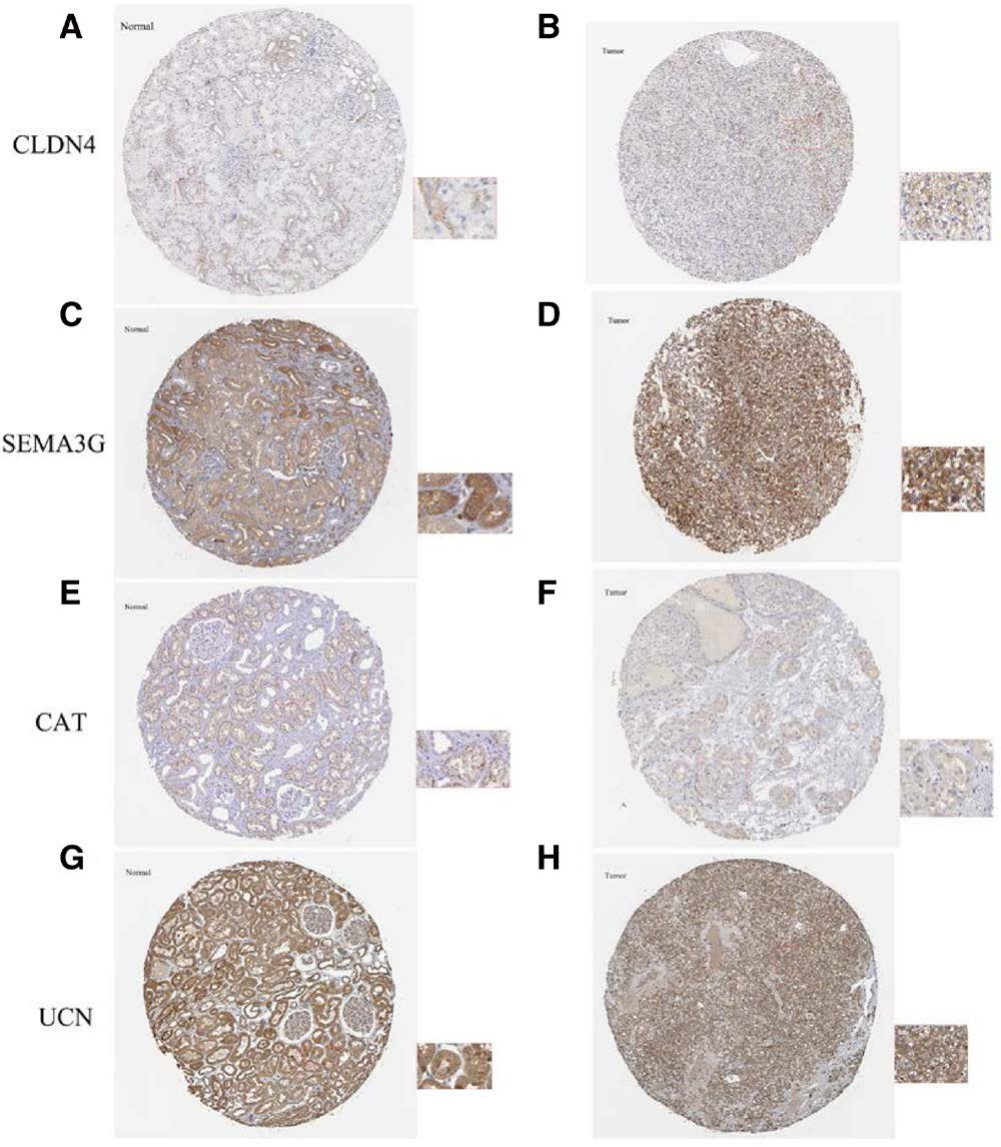
The expression of IRDEGs verified based on HPA database. (A–H) Immunohistochemistry (IHC) showed the expression levels of CLDN4, SEMA3G, CAT, UCN in normal kidney tissue and ccRCC. CAT = catalase, ccRCC = clear cell renal cell carcinoma, CLDN4 = claudin 4, IRDEG = immune-related differentially expressed gene, SEMA3G = semaphorin 3G, UCN = urocortin.

The transcriptional and survival data of ccRCC patients were divided into 3 groups: training, testing, and overall (Table 3). Subsequently, each group was further divided into high- and low-risk groups based on the median risk score. The risk scores for the training, testing, and overall groups exhibited a negative association with patient survival (Fig. 5A–F). A discernible pattern in the heatmap suggests that the genes CLDN4, SEMA3G, and CAT are highly expressed in the low-risk group, which insinuates their potential role as protective prognostic factors. On the contrary, the UCN gene displays high expression in the high-risk group, indicating its probable contribution as a negative prognostic factor (Fig. 5G–I). Notably, patients classified within the high-risk group manifested significantly diminished survival rates compared to those in the low-risk group (Fig. 5J–L). The area under the receiver operating characteristic (ROC) curve (area under the curve) for 1, 3, and 5 years was all greater than 0.70 (Fig. 5M–O). The conclusions drawn from the overall group (Fig. 5C, F, I, L, and O) were correspondingly validated in both the training group (Fig. 5A, D, G, J, and M) and the testing group (Fig. 5B, E, H, K, and N). Prognostic analyses were also performed based on an external KM plotter database, which showed that the protective prognostic factors CLDN4, SEMA3G, CAT, and the unfavorable prognostic factor UCN were strongly correlated with OS (*P* < .001) (Fig. 6A–D). Significantly, we juxtaposed the expression of these 4 pivotal genes and the constituent signatures between normal kidney tissues and renal cancers using gene chip data. The results indicated differential expression (*P* < .05) in both single-gene and multi-gene signatures. Notably, these multi-gene signatures also exhibited differential expression between metastatic cancer and normal kidney tissue (*P* < .05) (Fig. 6E–I). Using the HPA database to assess protein expression levels, we observed positive staining for CLDN4, SEMA3G, CAT, and UCN in the nuclei of ccRCC compared with normal tissues (Fig. 7A–H).

**Table 3:** Distribution of the number of different clinical characteristics in the training, testing, and overall groups.

Covariates	Type	Training	Testing	Overall	*P* value
Age	>65	108 (31.4%)	67 (38.95%)	175 (33.91%)	.1072
Age	≤65	236 (68.6%)	105 (61.05%)	341 (66.09%)	
Gender	FEMALE	122 (35.47%)	56 (32.56%)	178 (34.5%)	.5778
Gender	MALE	222 (64.53%)	116 (67.44%)	338 (65.5%)	
Grade	G1–2	158 (45.93%)	75 (43.6%)	233 (45.16%)	.6408
Grade	G3–4	180 (52.33%)	95 (55.23%)	275 (53.29%)	
Grade	Unknow	6 (1.74%)	2 (1.16%)	8 (1.55%)	
Stage	Stage I–II	206 (59.88%)	107 (62.21%)	313 (60.66%)	.7653
Stage	Stage III–IV	135 (39.24%)	65 (37.79%)	200 (38.76%)	
Stage	Unknow	3 (0.87%)	0 (0%)	3 (0.58%)	
T	T1–2	219 (63.66%)	112 (65.12%)	331 (64.15%)	.8203
T	T3–4	125 (36.34%)	60 (34.88%)	185 (35.85%)	
M	M0	266 (77.33%)	143 (83.14%)	409 (79.26%)	.3816
M	M1	56 (16.28%)	23 (13.37%)	79 (15.31%)	
M	Unknow	22 (6.4%)	6 (3.49%)	28 (5.43%)	
N	N0	163 (47.38%)	67 (38.95%)	230 (44.57%)	.5463
N	N1	13 (3.78%)	3 (1.74%)	16 (3.1%)	
N	Unknow	168 (48.84%)	102 (59.3%)	270 (52.33%)	

The comparison among groups was statistically significant, *P* < .05.

### 3.4. Independent prognostic analysis of the prognostic model

**Figure 8.**
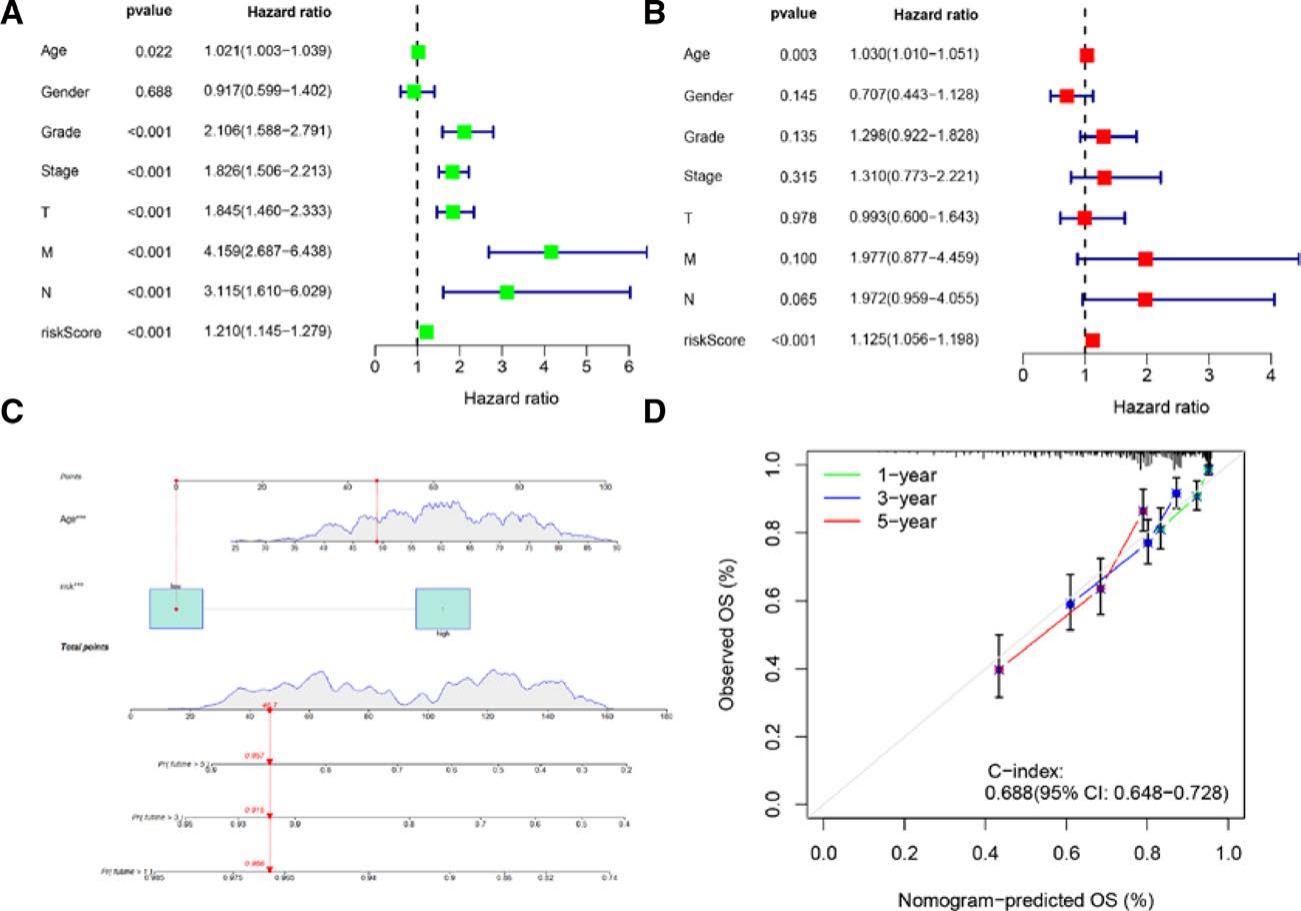
Assessment of the independent prognostic value of the risk model. Univariate (A) and multivariate (B) Cox regression analyses of risk score and clinical characteristics. (C) The nomogram combining risk score and age to predict OS at 1, 3, and 5 years for ccRCC patients. (D) Calibration curves. ***P < .001; **P < .001; *P < .05. ccRCC = clear cell renal cell carcinoma, OS = overall survival.

The study utilized clinical data from TCGA-KIRC to examine the relationship between risk scores and clinical characteristics with ccRCC prognosis, employing both univariate and multivariate Cox regression analyses. Our findings suggest that the risk model developed in this study independently predicts the prognosis of ccRCC (Fig. 8A and B). Furthermore, we developed a nomogram using 2 independent prognostic indicators, risk score and age. The nomogram assigns a total score to each patient based on their prognostic parameters (Fig. 8C), where a higher total score indicates a poorer prognosis. The calibration curve demonstrated the reliability of the nomogram in predicting prognostic performance (Fig. 8D).

### 3.5. Exploring the difference of immune infiltrating cells

**Figure 9.**
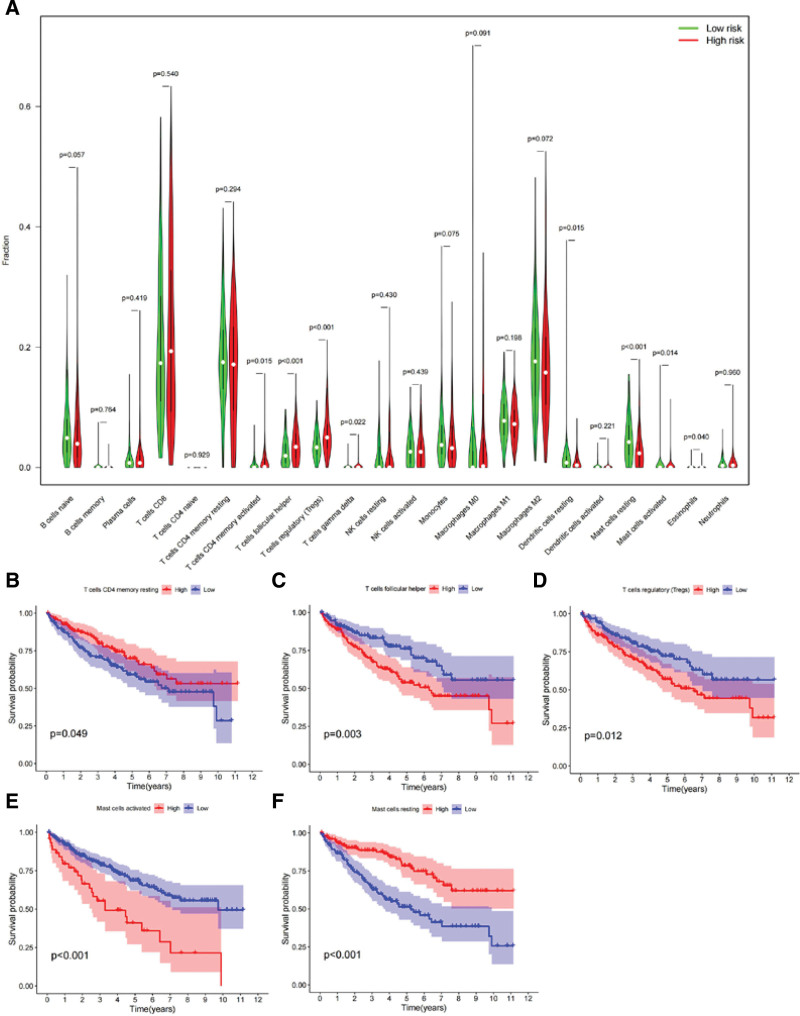
Immune infiltration and prognosis analysis in ccRCC patients. (A) Violin plots comparing 22 immune cell types between high-risk and low-risk groups of ccRCC patients. (B–F) Kaplan–Meier survival curves of immune cell infiltration levels and overall survival in ccRCC patients. ccRCC = clear cell renal cell carcinoma.

To investigate the potential relationship between the immune-related risk prognostic model and immune cell populations in ccRCC patients, we utilized the CIBERSORT algorithm to compare immune infiltration. In the high-risk group, activated CD4 memory T cells, follicular helper T cells, regulatory T cells (Tregs), and gamma delta T cells were the predominant cell types, while in the low-risk group, resting dendritic cells, resting and activated mast cells, and eosinophils were the main populations (Fig. 9A). Prognostic analysis of immune cell infiltration in ccRCC patients revealed that infiltration of resting CD4 memory T cells and resting mast cells was positively correlated with overall survival, whereas infiltration of follicular helper T cells, Tregs, and activated mast cells was negatively correlated with survival (Fig. 9B–F).

### 3.6. TMB and prognostic analysis

**Figure 10.**
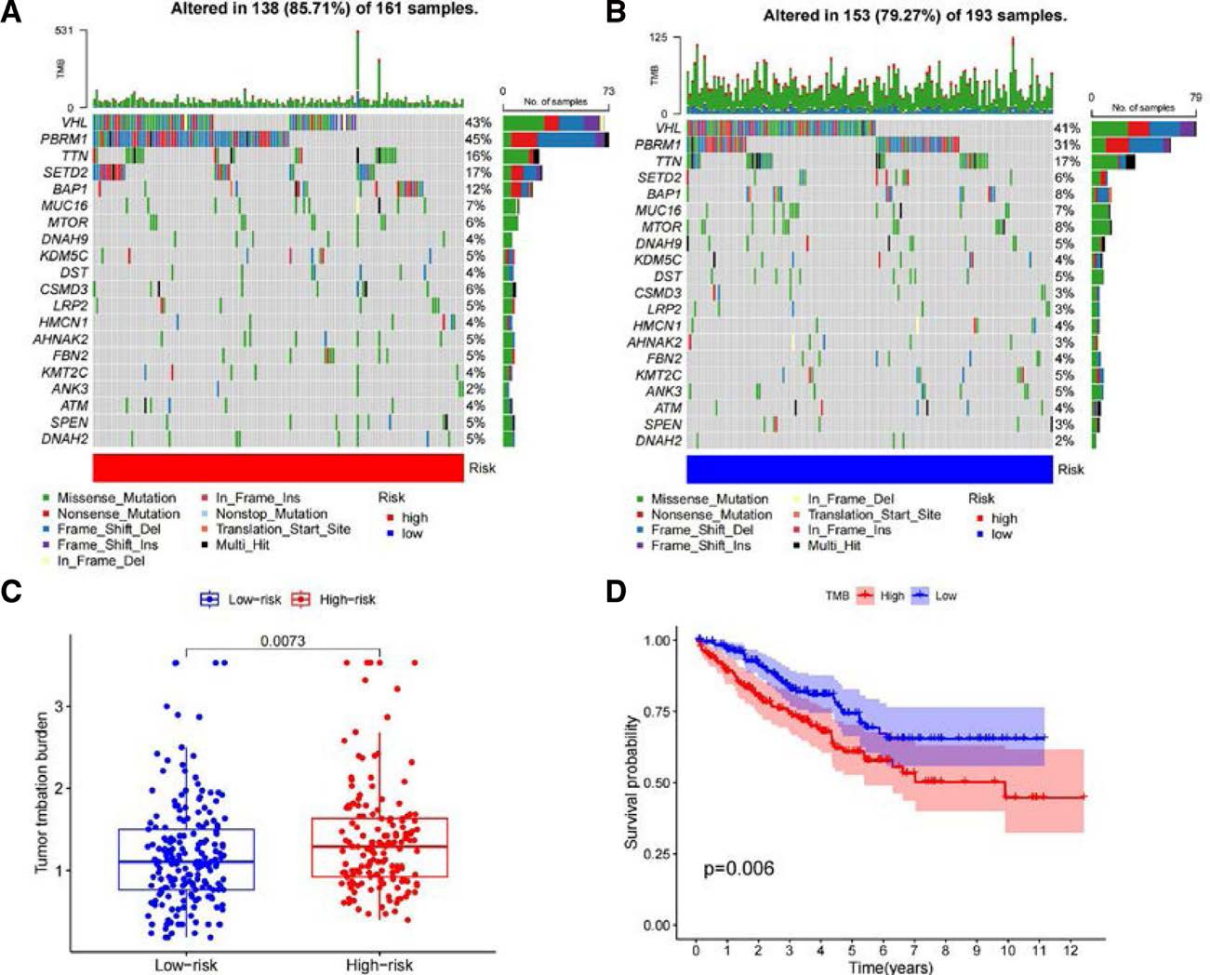
Analysis of somatic mutations in high- and low-risk groups. (A) Genes with the top 20 mutation frequencies in the high-risk group. (B) Genes with the top 20 mutation frequencies in the low-risk group. (C) Differences in tumor mutation burden (TMB) between high- and low-risk groups. (D) Kaplan–Meier curve of TMB in high- and low-risk groups and overall survival.

To determine the differences in cancer-associated gene mutations between high- and low-risk groups, we generated a mutation waterfall plot for each group (Fig. 10A and B). The top 5 genes with the highest mutation frequencies were consistent in both groups: VHL, PBRM1, TTN, SETD2, and BAP1. Genes that showed significantly different mutation frequencies between the high- and low-risk groups were the tumor suppressor gene PBRM1 (45% vs 31%) and the protein coding gene SETD2 (17% vs 6%). Furthermore, the overall TMB value was higher in the high-risk group compared to the low-risk group (Fig. 10C), and the overall survival rate was markedly lower in the TMB-H group compared to the TMB-L group (Fig. 10D). These findings suggest that TMB may serve as a potential prognostic marker for ccRCC patients.

### 3.7. IPS and immune checkpoint inhibitors analysis

**Figure 11.**
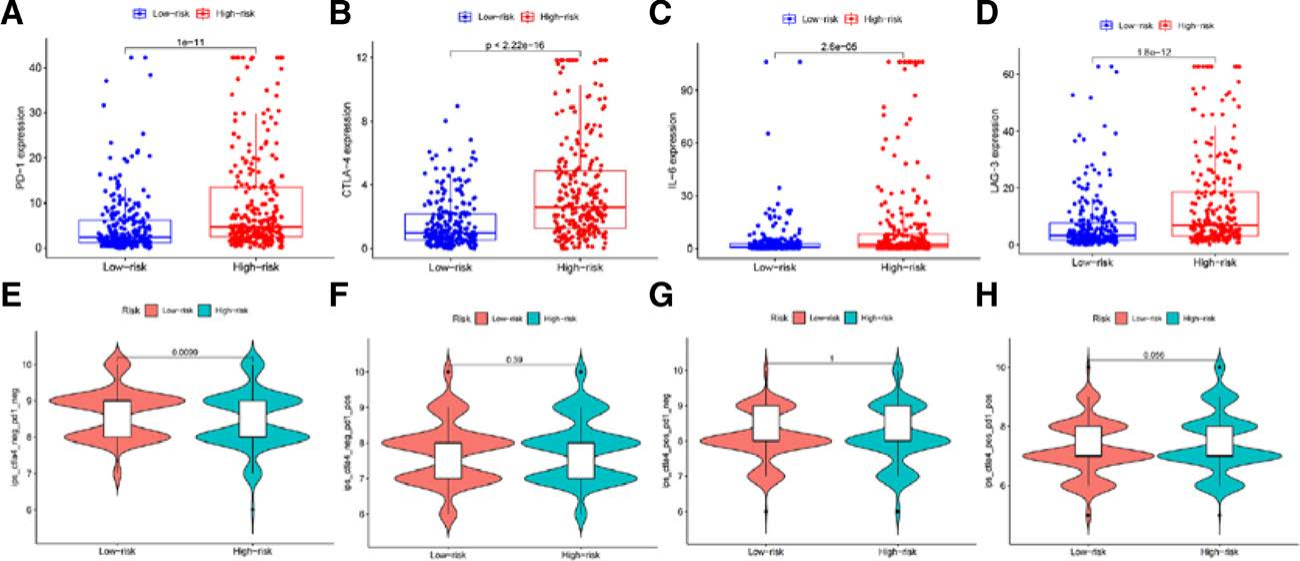
Sensitivity analysis of high- and low-risk groups of ccRCC patients to immune inhibitors. (A–D) Expression levels of PD-1, CTLA-4, IL-6, and LAG-3 immune checkpoints in high- and low-risk groups. (E–H) The relationship between the Immunophenoscore (IPS) of high-risk and low-risk groups of ccRCC patients and PD-1 and CTLA-4 immune checkpoint inhibitors. ccRCC = clear cell renal cell carcinoma, CTLA-4 = cytotoxic T lymphocyte-associated antigen-4, PD-1 = programmed cell death protein 1.

To ascertain the prognostic impact of immunotherapy across high-risk and low-risk cohorts, we charted the expression levels of PD-1, CTLA-4, IL-6, and LAG3 via scatter plots for both groups. The findings revealed a notably higher sensitivity to the quartet of immune checkpoints in the high-risk cohort compared to the low-risk counterpart (*P* < .001) (Fig. 11A–D). Leveraging previously published data, we observed a significant decrease in IPS-PD-1 (−) CTLA-4 (−) for the high-risk group versus the low-risk group, in the context of PD-1 and CTLA-4 co-administration (*P* < .01) (Fig. 11E). Regrettably, the mating data for IPS-PD-1-CTLA-4 did not exhibit significant disparities in the outcomes (Fig. 11F–H). This observation implicitly highlights the high-risk group’s increased susceptibility to immune checkpoint inhibitors, thereby corroborating our hypothesis that the aforementioned immune checkpoint inhibitors may be efficaciously employed within high-risk groups.

## 4. Discussion

Presently, the primary therapeutic approach for individuals diagnosed with limited-stage ccRCC involves surgical resection of localized lesions. However, the postoperative mortality rate is often higher in elderly patients and those with a high degree of malignancy.^[33]^ The effectiveness of chemotherapy and targeted therapy in patients with metastatic ccRCC is not optimistic. Existing studies have demonstrated that various cellular components in the TME can be targeted for anticancer therapy.^[8]^ The aggregation, expansion, and potent anticancer activity of cytotoxic T lymphocytes in the TME contribute to immunotherapy.^[34]^

In conclusion, our study successfully established a prognostic model for ccRCC immune-related risk, identifying CLDN4, SEMA3G, CAT, and UCN as principal genes. Subsequent differential expression validation, performed utilizing external databases, further underscored the high clinical prognostic value of these 4 key genes and the proposed model, particularly in forecasting renal cancer progression and metastasis. CLDN4 is a target of the Claudins protein family, and nuclear translocation of CLDN4 enhances the epithelial-mesenchymal transition phenotype, resulting in the replacement of CLDN4 tightly linked with Yes-associated protein (YAP) and zonular atresin (ZO-1), forming a nuclear translocation complex, which is one of the mechanisms of renal cancer formation.^[35]^ Research by Hu et al^[36]^ demonstrates that CLDN4 suppression in Acute Myeloid Leukemia leads to decreased activity of AKT and ERK1/2. This signal transduction pathway is situated downstream of EGFR activation. Previous foundational studies on cancer have established that the EGFR pathway and AKT signaling play significant roles in influencing immune infiltration and the immune microenvironment within the PD-1/PD-L1 pathway.^[37,38]^ Studies have shown that the overexpression or deletion of Claudins proteins is a key factor in malignant tumor formation, as confirmed in various cancers, including ovarian cancer and breast cancer. Additionally, CLDN4 has been recognized as an independent prognostic factor for gastric cancer.^[39–42]^ SEMA3G (Semaphorin 3G), a gene that encodes a glycoprotein and is involved in tumor development and angiogenesis, has also been implicated in this context.^[43]^ SEMA3G can negatively regulate the signal transduction of vascular endothelial growth factor, regulate cell adhesion, and induce apoptosis.^[44]^ In gliomas, SEMA3G signaling is transmitted to the PI3K/Akt pathway via neuropilin receptors, Plexins-A, which stimulates cell growth, migration, and invasion.^[45]^ Data derived from murine models provides further insight, revealing that the PI3K-Akt-mTOR pathway is capable of controlling deactivation of immune suppression pathways and augmenting innate immune properties integral to tumor immune surveillance.^[46]^ The protein-encoding gene, CAT (Catalase), distinguished by its superior biocompatibility and potent catalytic capacity, shields cells from the deleterious effects of hydrogen peroxide, thereby mitigating tumor hypoxia. Catalase can manipulate tumor oxygenation and further modulate the TME via immune factors, thereby reinforcing immune anti-tumor properties.^[47,48]^ UCN (Urocortin) is a stress-related corticotropin-releasing factor that exerts its effects on cell proliferation through autocrine and paracrine mechanisms. Evidence suggests that UCN stimulates kinase phosphorylation, promoting breast cancer proliferation.^[49]^ UCN1 has been observed to enhance the expression of ICAM1 through 2 distinct pathways: the cPLA2-NF-κB pathway and the cPLA2-COX2-PGE2-PKA-CREB pathway.^[50]^ ICAM1 mediates the interaction between cancer cells and T-cells, which is pivotal in forming a T-cell deficient TME. Evidence from lung cancer models suggests that ICAM1 present on cancer cells plays a coordinating role in immune anti-tumor responses.^[51]^ In a murine model, UCN2 has been demonstrated to promote the initiation and progression of prostate tumors through its inhibitory effect on apoptosis.^[52]^ Currently, there is a lack of research on the prognostic relevance of these core genes in ccRCC immunotherapy, which may be a direction for further investigation and expansion.

The survival outcomes in the risk groups are in accordance with the findings reported by Zhou, based on the model constructed using the aforementioned genes.^[53]^ Moreover, the findings from the multivariate regression analysis supported the notion that the risk score can serve as an independent prognostic factor, accounting for clinical characteristics such as clinical outcome, age, tumor stage, and histological grade. Additionally, ccRCC, being an immunogenic tumor, is known to induce immune dysfunction through the infiltration of immunosuppressive cells in the TME.^[54]^ Immune cell infiltration analysis revealed that high infiltration of regulatory T cells, follicular helper T cells, and activated mast cells was associated with poor prognosis, corroborating the findings of Pan,^[55]^ who also verified the correlation between immune infiltration of regulatory T cells and follicular helper T cells with prognosis. Fu’s finding^[56]^ showed that tumor-infiltrating mast cells were positively correlated with overall survival in non-metastatic ccRCC.

Currently, TMB has gained recognition as a promising biomarker for prognostication and prediction of response to immunotherapy in diverse cancer types. ccRCC patients account for a significant proportion of kidney cancer cases and are often diagnosed at late stages with limited treatment options. The predictive value of TMB in immunotherapy response in renal cancer remains inconclusive.^[57,58]^ Our somatic mutation profile revealed that VHL had the highest frequency in both high- and low-risk groups, while PBRM1 and SETD2 showed substantial differences in mutation frequency between the 2 groups. VHL is an important tumor suppressor gene, and although VHL inactivation is common in various tumor types, ccRCC exhibits significant heterogeneity in VHL mutations.^[2,59]^ The VHL protein complex facilitates the recruitment of the E3 ligase complex, leading to ubiquitination and subsequent degradation of hypoxia-inducible factor α (HIFα). Loss of VHL results in the accumulation of HIFα, leading to activation of key oncogenic pathways of HIF.^[60,61]^ Mutational inactivation of VHL has been shown to preserve tumor response to PD-1 immunotherapy by regulating HIF-1α and HIF-2α.^[62]^ PBRM1 is a tumor suppressor gene encoding BAF180 protein, and studies have reported that 28% to 55% of ccRCC patients have PBRM1 mutations driven by VHL mutations.^[63]^ PBRM1 mutations have been associated with immunotherapy response and survival rate in advanced renal cell carcinoma.^[64]^ SETD2 is a histone lysine methyltransferase, and genomic analysis in ccRCC has revealed that SETD2 mutations are positively associated with metastasis, and loss of SETD2 leads to amplified transcription of oncogenic drivers.^[65]^ A pan-cancer analysis conducted by Pornpimol^[66]^ demonstrated that transcription of genes related to immune activity was up-regulated in patients with SETD2-mutated tumors, which was associated with favorable clinical outcomes. The IPS analysis disclosed an enhanced sensitivity towards the key immune checkpoints – PD-1, CTLA-4, IL-6, and LAG3 – in the high-risk group. Despite existing data failing to conclusively determine if the co-inhibition or elimination of PD-1 and CTLA-4 would influence patient prognosis significantly, the emergent sensitivity of these immune checkpoints in the risk model carves a new path for advancing immunotherapy for ccRCC.

Nevertheless, our study is not without limitations. Firstly, we did not conduct basic experiments to confirm the association between the 4 immune genes and immune infiltration in ccRCC. Additionally, the availability of clinical data was limited, impeding the validation of the predictive efficacy of the immune-related risk prognosis model.

## 5. Conclusion

CLDN4, SEMA3G, CAT, and UCN have been identified as potential prognostic markers for ccRCC. A model based on these 4 genes has the potential to predict immune efficacy.

## Acknowledgments

The authors thank all the participants for their cooperation.

## Author contributions

**Conceptualization:** Ronghui Chen.

**Data curation:** Ronghui Chen, Jun Wu.

**Formal analysis:** Shan Liu.

**Investigation:** Yefeng Sun, Guozhi Liu, Lin Zhang.

**Software:** Yefeng Sun, Guozhi Liu.

**Validation:** Qing Yu, Juan Xu.

**Visualization:** Shan Liu, Lingxin Meng.

**Writing – original draft:** Ronghui Chen, Jun Wu.

**Writing – review & editing:** Ronghui Chen, Jun Wu, Lingxin Meng.
